# Crystal structure of the hydroxylaminopurine resistance protein, YiiM, and its putative molybdenum cofactor-binding catalytic site

**DOI:** 10.1038/s41598-018-21660-y

**Published:** 2018-02-19

**Authors:** Byeol Namgung, Jee-Hyeon Kim, Wan Seok Song, Sung-il Yoon

**Affiliations:** 1 Division of Biomedical Convergence, College of Biomedical Science, Kangwon National University, Chuncheon, 24341 Republic of Korea; 2 Institute of Bioscience and Biotechnology, Kangwon National University, Chuncheon, 24341 Republic of Korea

## Abstract

The molybdenum cofactor (Moco) is a molybdenum-conjugated prosthetic group that is ubiquitously found in plants, animals, and bacteria. Moco is required for the nitrogen-reducing reaction of the Moco sulfurase C-terminal domain (MOSC) family. Despite the biological significance of MOSC proteins in the conversion of prodrugs and resistance against mutagens, their structural features and Moco-mediated catalysis mechanism have not been described in detail. YiiM is a MOSC protein that is involved in reducing mutagenic 6-N-hydroxylaminopurine to nontoxic adenine in bacteria. Here, we report two crystal structures of YiiM: one from Gram-positive *Geobacillus stearothermophilus* (gsYiiM) and the other from Gram-negative *Escherichia coli* (ecYiiM). Although gsYiiM and ecYiiM differ in oligomerization state and protein stability, both consist of three structural modules (a β-barrel and two α-helix bundles) and feature a cavity surrounded by the three modules. The cavity is characterized by positive electrostatic potentials and high sequence conservation. Moreover, the ecYiiM cavity houses a phosphate group, which emulates a part of Moco, and contains a highly reactive invariant cysteine residue. We thus propose that the cavity is the catalytic site where Moco binds and the substrate is reduced. Moreover, our comparative structural analysis highlights the common but distinct structural features of MOSC proteins.

## Introduction

Molybdenum in living organisms, except for that in nitrogenase, is usually bound to molybdopterin (MPT) to form a molybdenum cofactor (Moco) and exert catalytic activity^1,2^. More than 50 cellular enzymes require Moco to catalyze redox reactions involved in carbon, sulfur, and nitrogen cycles. Moco-dependent enzymes were traditionally classified into three groups depending on the types of Moco modification and conjugation, including the sulfite oxidase family, the xanthine oxidase family, and the dimethyl sulfoxide reductase family^3^. In addition to the conventionally classified groups, a new family of Moco-binding enzymes was identified and named as the Moco sulfurase C-terminal domain (MOSC) family due to its sequence homology with the C-terminal domain of Moco sulfurase^4^. MOSC family members do not share any sequence similarity with the three existing Moco families.

MOSC proteins are ubiquitously found in bacteria and animals. However, only several MOSC family members, including human mitochondrial amidoxime reducing component (mARC) and *Escherichia coli* YiiM and YcbX, have been functionally characterized. mARC, YiiM, and YcbX are involved in the reduction of N-hydroxylated substrates. mARC detoxifies mutagenic N-hydroxylated base analogs, such as 6-N-hydroxylaminopurine (HAP), and activates N-hydroxylated amidoxime prodrugs as a redox system in concert with NADH-cytochrome b_5_ reductase (Cytb_5_R) and cytochrome b_5_ (Cytb_5_)^5–8^. In analogy to nitrate reductase, electrons were proposed to flow from NADH to the mARC Moco through the Cytb_5_R flavin and Cytb_5_ heme, resulting in mARC activation for the reduction of N-hydroxylated substrates^9^. YiiM and YcbX commonly contribute to resistance against toxic N-hydroxylated base analogs in *E. coli*^10^. YcbX drives the nitrogen-reducing reaction for HAP along with CysJ, which reduces flavin using NADPH^11,12^. Thus, CysJ is expected to deliver electrons from NADPH through flavin to the iron-sulfur cluster in the C-terminal Fer domain of YcbX. Next, YcbX would transfer electrons from the iron-sulfur cluster through Moco to the N-hydroxylated substrates. Notably, *Vibrio cholerae* and *Vibrio parahaemolyticus* contain a single protein (VCA0924 and VPA0411, respectively) that includes all the domains of *E. coli* YcbX and CysJ^11^. MOSC domains therefore function in collaboration with electron transfer proteins or domains. However, the electron transfer partner of YiiM has not been identified although YiiM, like YcbX, is involved in the detoxification of HAP.

MOSC proteins play a key role in the Moco-dependent reduction of N-hydroxylated mutagens or prodrugs as a final electron transfer enzyme in the nitrogen-reducing system. Despite the functional importance of Moco in MOSC-mediated enzymatic reactions, the structural mechanism of Moco-mediated catalysis in the MOSC family remains unknown. In the MOSC family, one structure of apo form of YiiM was published solely through a structural genomics project, and it did not provide any structural insights into the enzymatic reaction of the MOSC family beyond a brief description of the overall structure^13^. To provide insights into the catalytic mechanism of MOSC enzymes, we report two crystal structures of YiiM, one from Gram-positive *Geobacillus stearothermophilus* (gsYiiM) and the other from Gram-negative *E. coli* (ecYiiM). Based on the comparative analyses of structural and biophysical data, we present the common and distinct features of YiiM enzymes and propose the putative catalytic site that is located at a reactive invariant cysteine residue.

## Results and Discussion

### Overall structure of YiiM

**Figure 1:**
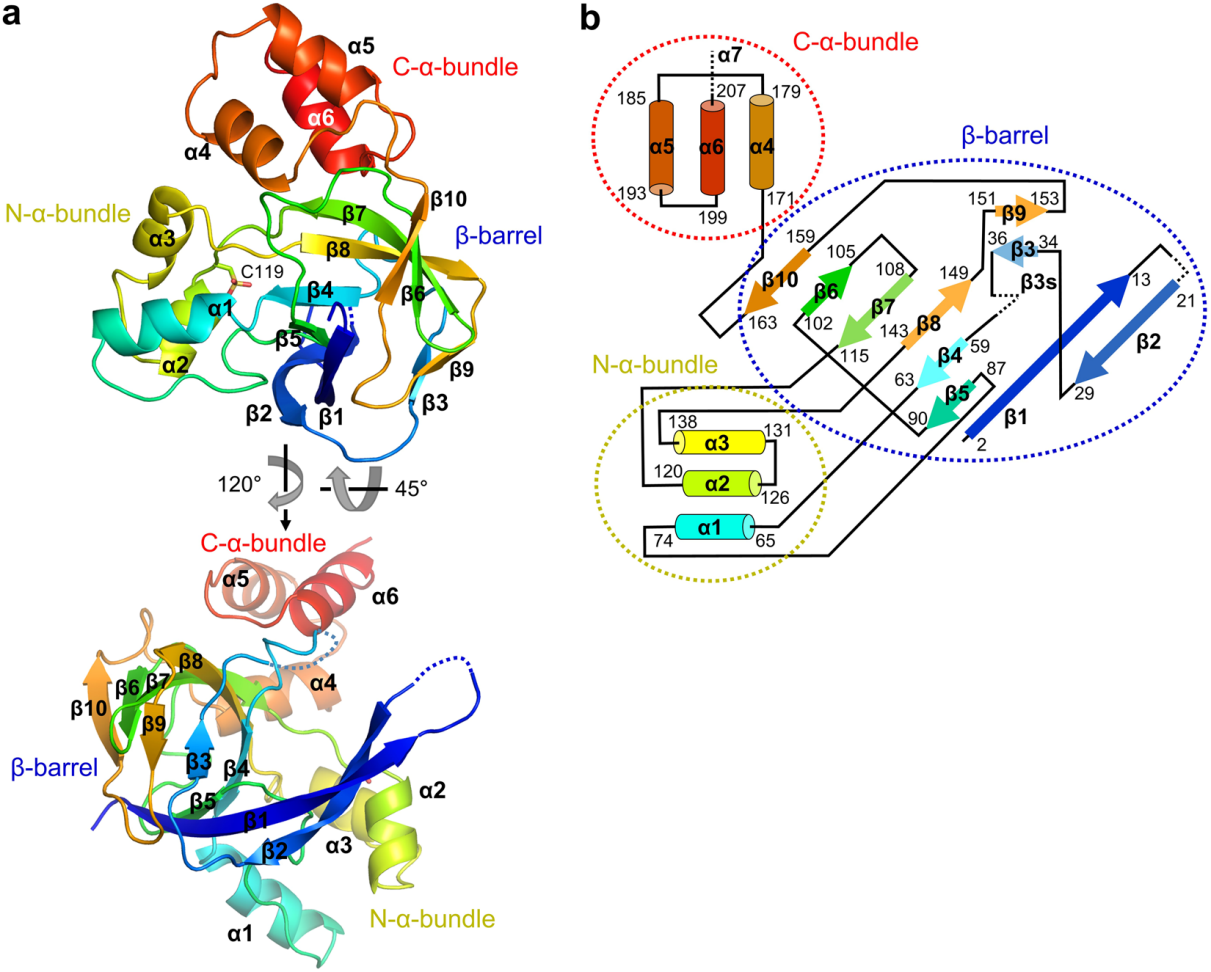
Overall structure of gsYiiM. (**a**) The crystal structure of gsYiiM. The gsYiiM structure is shown as rainbow-colored ribbons ranging from blue at the N-terminus to red at the C-terminus. Dotted lines represent disordered regions that were not built in the gsYiiM structure. (**b**) Topological diagram that exhibits the secondary structures of gsYiiM. The α-helices and β-strands of gsYiiM are represented by rods and arrows, respectively. ecYiiM contains additional secondary structures (β3s and α7), which are labeled in the figure without rod or arrow representation.

For the structural study of YiiM, recombinant gsYiiM protein was expressed in *E. coli* BL21 (DE3) cells, purified by two purification steps, and crystallized in drops containing PEG 4000. The crystal structure of gsYiiM was determined by molecular replacement and refined to 2.00 Å resolution (Table 1). The gsYiiM structure consists of ten β-strands and six α-helices and folds into a triangular shape that can be divided into three distinct structural modules, namely, a β-barrel, an N-terminal α-helix bundle (N-α-bundle), and a C-terminal α-helix bundle (C-α-bundle) (Fig. 1). Eight of the ten β-strands are organized into the β-barrel in the order of the β2, β1, β5, β4, β8, β7, β6, and β10 strands, with the β4, β7 and β8 strands at the center of the gsYiiM structure. The two remaining β-strands, β3 and β9, cover one open end of the β-barrel as a lid and form one vertex of the gsYiiM triangle. The β-barrel is decorated with the N-α-bundle (α1, α2, and α3) and the C-α-bundle (α4, α5, and α6), each of which forms one vertex of the gsYiiM triangle. Sequences corresponding to the β-barrel and N-α-bundle modules of YiiM are conserved in MOSC family members, suggesting that the two modules form the canonical structure of the MOSC domain. In contrast, the C-α-bundle is exclusively observed in YiiM.

**Table 1:** Crystallographic statistics of the YiiM structures.

	gsYiiM	**e**cYiiM^Pi^
**Data collection**		
Space group	P2_1_2_1_2_1_	P2_1_2_1_2_1_
Cell parameters		
a (Å)	46.42	56.16
b (Å)	51.53	84.84
c (Å)	94.21	107.63
Wavelength (Å)	1.00004	0.98000
Resolution (Å)	30.00–2.00	30.00–2.85
Highest resolution (Å)	2.07–2.00	2.90–2.85
No. observations	90,078	78,041
No. unique reflections	15,858	12,483
R_merge_ (%)^a^	6.6 (50.0)^b^	12.2 (43.0)^b^
I/sigma(I)	38.9 (4.8)^b^	14.7 (2.3)^b^
Completeness (%)	99.7 (100.0)^b^	99.7 (96.6)^b^
Redundancy	5.7 (5.8)^b^	6.3 (5.6)^b^
**Refinement**		
Resolution (Å)	30.00–2.00	30.00–2.85
No. of reflections (work)	14,960	11,838
No. of reflections (test)	783	599
R_work_ (%)^c^	18.5	20.1
R_free_ (%)^d^	22.3	24.2
No. atoms		
Protein	1,494	3,193
Ligands (phosphate)	0	10
Water	49	0
Average B-value (Å^2^)	38.9	51.6
RMSD bonds (Å)	0.015	0.012
RMSD angles (°)	1.44	1.28
Ramachandran^e^ (favored)	98.9%	99.0%
(outliers)	0.0%	0.0%

^a^R_merge_ = Σ_hkl_Σ_i_ | I_i_(hkl) − < I(hkl) > |/Σ_hkl_Σ_i_ I_i_(hkl).

^b^Numbers in parenthesis were calculated from the data of the highest resolution shell.

^c^R_work_ = Σ| |F_obs_| − |F_calc_| |/Σ|F_obs_| where F_calc_ and F_obs_ are the calculated and observed structure factor amplitudes, respectively.

^d^R_free_ = as for R_work_, but 5% of the total reflections were chosen at random and omitted from refinement.

^e^Calculated using MolProbity (http://molprobity.biochem.duke.edu).

### Putative catalytic site of YiiM

**Figure 2:**
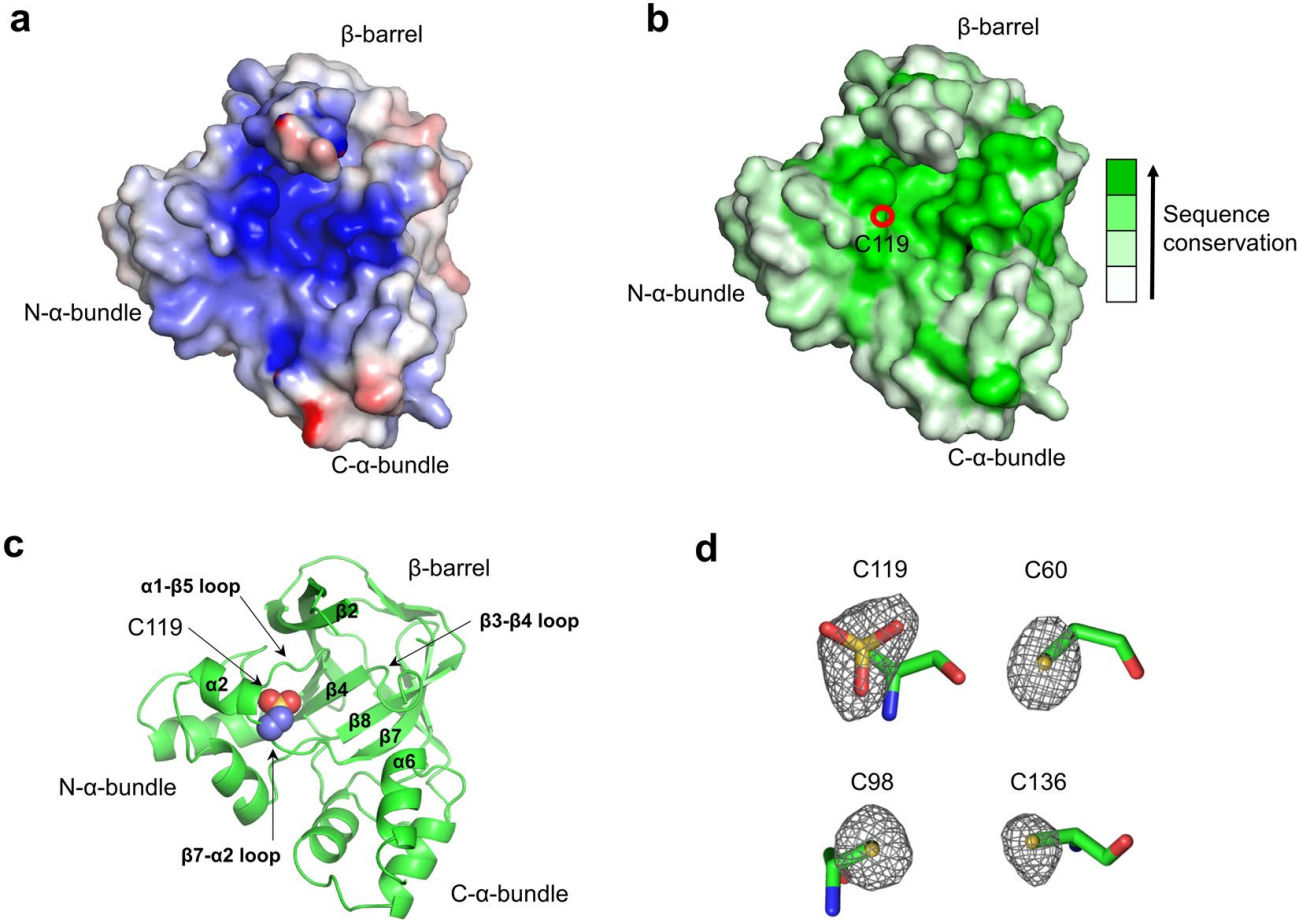
gsYiiM cavity as a putative catalytic site. (**a**) Electrostatic potential surface representation of the gsYiiM structure. The figure is viewed from the back of gsYiiM in the upper panel of Fig. 1a. (**b)** Surface representation of the gsYiiM structure that is color-coded by sequence conservation. The sequence conservation was calculated using the ConSurf server^27^. The invariant cysteine residue (C119) of gsYiiM is represented by a red circle. The orientation of gsYiiM in the figure is identical to that of gsYiiM in Fig. 2a. (**c**) Oxidized cysteine residue 119 (sulfur, a yellow sphere; oxygen, red spheres; carbon, light blue spheres) in the gsYiiM structure (green ribbons). The orientation of gsYiiM in the figure is identical to Fig. 2a. (**d**) Fo-Fc omit electron density map (gray wires shown in a 3.5σ level) for the sulfur atoms of four cysteine residues (C60, C98, C119, and C136; sticks) in the gsYiiM structure. The Sγ and Cβ atoms of the cysteine residues are vertically aligned.

**Figure 3:**
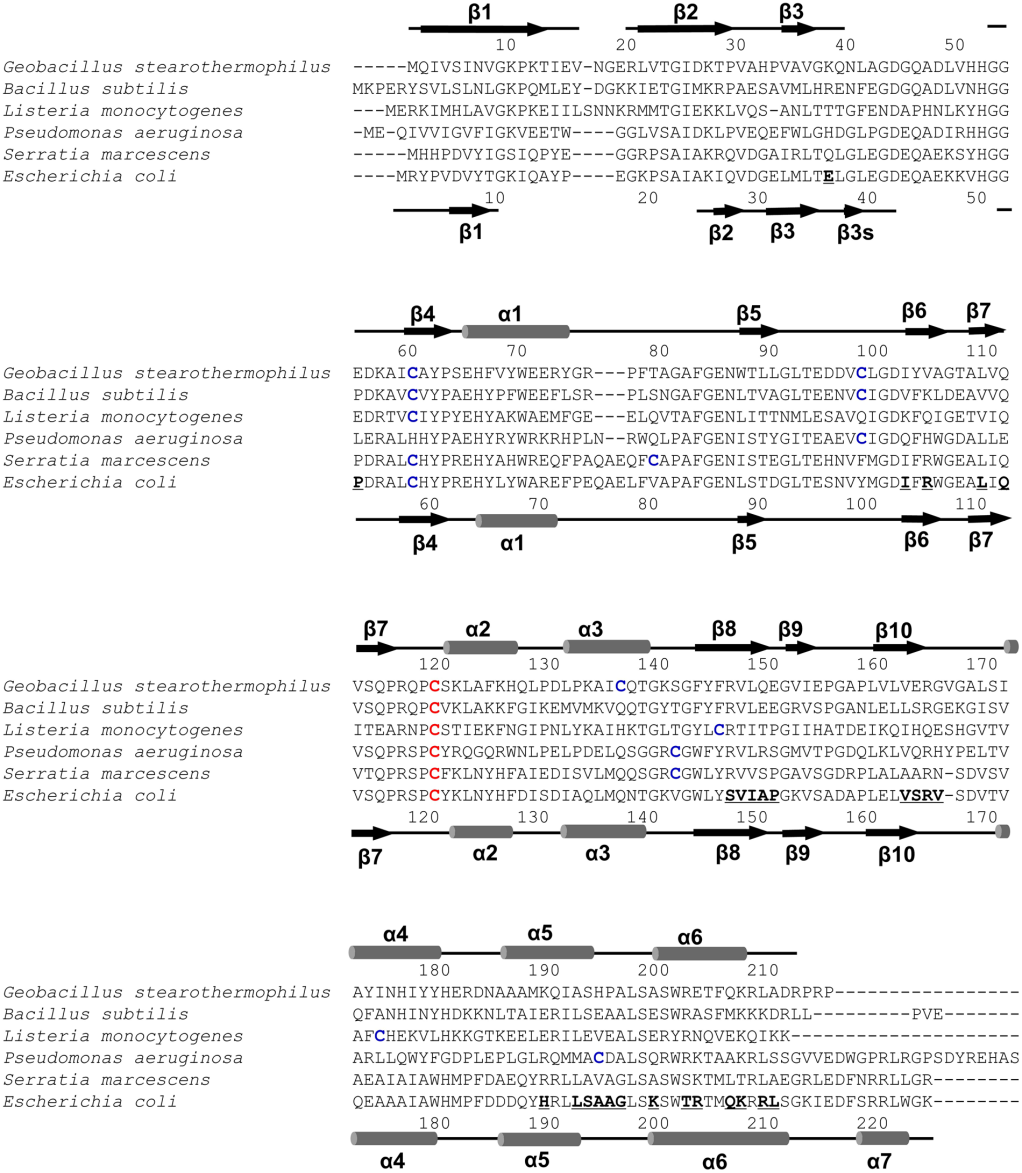
Sequence alignment of YiiM orthologs. The secondary structures of gsYiiM and ecYiiM are shown above the gsYiiM sequence and below the ecYiiM sequence, respectively (α-helix, rod; β-strand, arrow). The invariant cysteine residue (gsYiiM C119 and ecYiiM C120) of the MOSC family is colored in red, and the remaining cysteine residues are blue. The dimerization interface residues of ecYiiM observed in the ecYiiM^Pi^ structure are bold and underlined.

gsYiiM possesses a cavity in the middle of its triangular architecture (Fig. 2a,b,c). The bottom of the cavity is built with the β4, β8, and β7 strands and the α1-β5 loop, and the sidewall of the cavity is buttressed by β2, the β3-β4 loop, α6, the β7-α2 loop, and α2. Consistent with the Moco-binding sites resolved in sulfite dehydrogenase (PDB ID 2CA4) and nitrate reductase (PDB ID 2BIH), the surface of the gsYiiM cavity is characterized with positive electrostatic potentials, presumably to hold a negatively charged molecule, such as Moco (Fig. 2a)^14,15^. Moreover, the cavity residues of gsYiiM are highly conserved in YiiM orthologs, suggesting a critical role of the cavity in the enzymatic function of YiiM (Fig. 2b). The gsYiiM cavity holds an invariant cysteine residue (C119 in gsYiiM), which is absolutely conserved in the entire MOSC family (Figs 2b,c and 3)^4^. Unexpectedly, in the Fo-Fc electron density map of gsYiiM, the sulfur atom of C119 exhibited an atypical triangular shape rather than a regular spherical shape that was observed for those of the other three cysteine residues (C60, C98, and C136) of gsYiiM (Fig. 2d). Considering the unusual shape and size of the electron density, the C119 sulfur atom is highly likely to have been oxidized to a sulfonic acid. Thus, the C119 residue was built in the gsYiiM structure as a cysteine sulfonic acid with good refinement statistics (Fig. 2c). C119 seems to be the most reactive among the four cysteine residues of gsYiiM, allowing us to propose the function of the C119 residue in the catalytic reaction of YiiM. The positive electrostatic potentials that surround the C119 residue would lower the pK_a_ value of the sulfhydryl group at C119, and the deprotonated sulfur could readily make a nucleophilic attack on Moco for cofactor conjugation or on a substrate for the nitrogen-reducing process. Therefore, gsYiiM could employ the C119 residue as a Moco-conjugating residue or a catalytic residue. Taken together, we propose that the gsYiiM cavity that encompasses the reactive C119 residue functions as a putative catalytic site where the catalysis of YiiM occurs.

### Moco binding to the YiiM cavity

**Figure 4:**
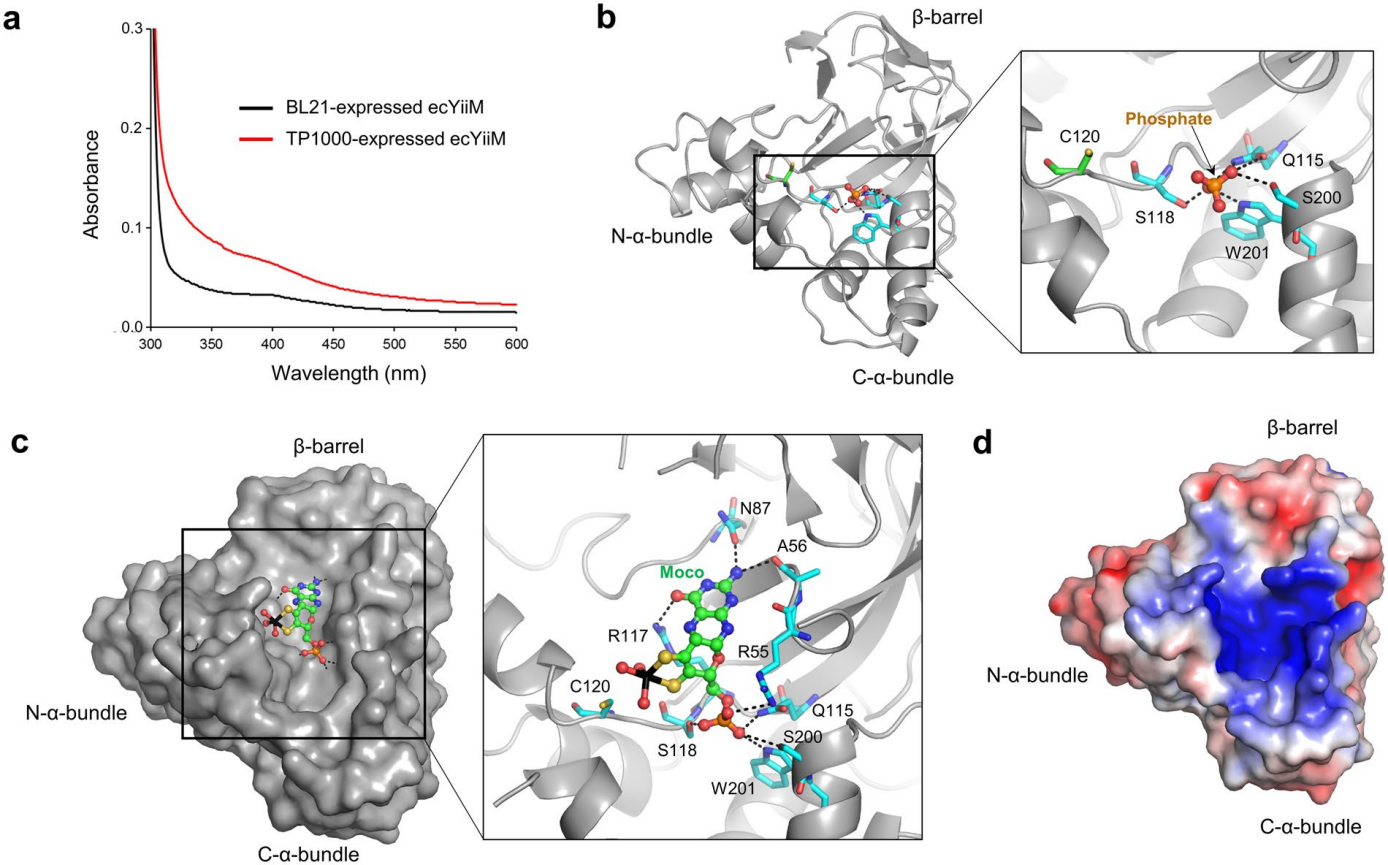
Putative Moco-binding site in the cavity of YiiM. (**a**) UV-visible absorption spectra for recombinant ecYiiM proteins (1.68 mg/ml) produced in *E. coli* BL21 and TP1000 cells. The absorption spectra were obtained using the Libra S80 instrument (Biochrom). (**b**) Phosphate ion located in the ecYiiM^Pi^ structure. The phosphate ion and the ecYiiM residues that bind it are shown as a ball-and-stick model (phosphorus, orange; oxygen, red) and sticks (carbon, cyan; oxygen, red; nitrogen, blue), respectively. The invariant cysteine residue (C120) of ecYiiM is depicted as sticks (sulfur, yellow; carbon, green; oxygen, red; nitrogen, blue). (**c**) A Moco molecule modeled in the ecYiiM structure (gray surface in the left figure; gray ribbons in the right figure). Moco is depicted as a ball-and-stick model (molybdenum, black; sulfur, yellow; phosphorus, orange; oxygen, red; nitrogen, blue). The Moco-binding residues of YiiM in the ecYiiM-Moco model are shown as sticks (sulfur, yellow; carbon, cyan; oxygen, red; nitrogen, blue) in the right figure. Hydrogen bonds are represented by dashed lines. The orientation of ecYiiM in the figure is identical to that of Fig. 4b. (**d**) Electrostatic potential surface representation of the ecYiiM^Pi^ structure. The cavity of ecYiiM is characterized with positive electrostatic potentials.

To empirically verify that YiiM binds Moco, molybdenum contents in YiiM proteins were analyzed by inductively coupled plasma mass spectrometry (ICP-MS). For the ICP-MS analysis, recombinant gsYiiM and ecYiiM proteins were produced in two different *E. coli* strains, BL21 (DE3) and TP1000. TP1000 cells have been reported to produce a high level of endogenous Moco molecules and are thus expected to enhance the relative ratio of a Moco-bound form of the expressed YiiM protein over an apo form compared to BL21 cells^16^. Indeed, the molybdenum contents of the TP1000-expressed YiiM proteins (16.4% and 11.4% for gsYiiM and ecYiiM, respectively) were substantially higher than those of the BL21-expressed proteins (0.2% and 0.3% for gsYiiM and ecYiiM, respectively), demonstrating that YiiM is a Moco-binding protein (Table 2). In addition to ICP-MS, the recombinant YiiM proteins were analyzed by absorption spectrometry. The TP1000-expressed ecYiiM protein displayed higher light absorbance at 300–600 nm compared to the BL21-expressed counterpart, suggesting the higher Moco content of the TP1000-expressed ecYiiM protein (Fig. 4a). In the spectra, a shoulder was observed between 350 nm and 400 nm as for other MOSC family proteins, including mARC and Moco sulfurase^7,17^.

**Table 2:** Molybdenum contents in recombinant gsYiiM and ecYiiM proteins produced in *E. coli* BL21 (DE3) and TP1000 cells.

gsYiiM (BL21)^a^	gsYiiM (TP1000)^a^	ecYiiM (BL21)^a^	ecYiiM (TP1000)^a^
0.002 ± 0.000^b^	0.164 ± 0.007^b^	0.003 ± 0.000^b^	0.114 ± 0.001^b^

^a^The parentheses indicate the strain of *E. coli* cells that was used for the recombinant protein expression.

^b^The molar content of molybdenum in 1 mole of YiiM protein (mean ± S.D) was determined by ICP-MS.

**Figure 5:**
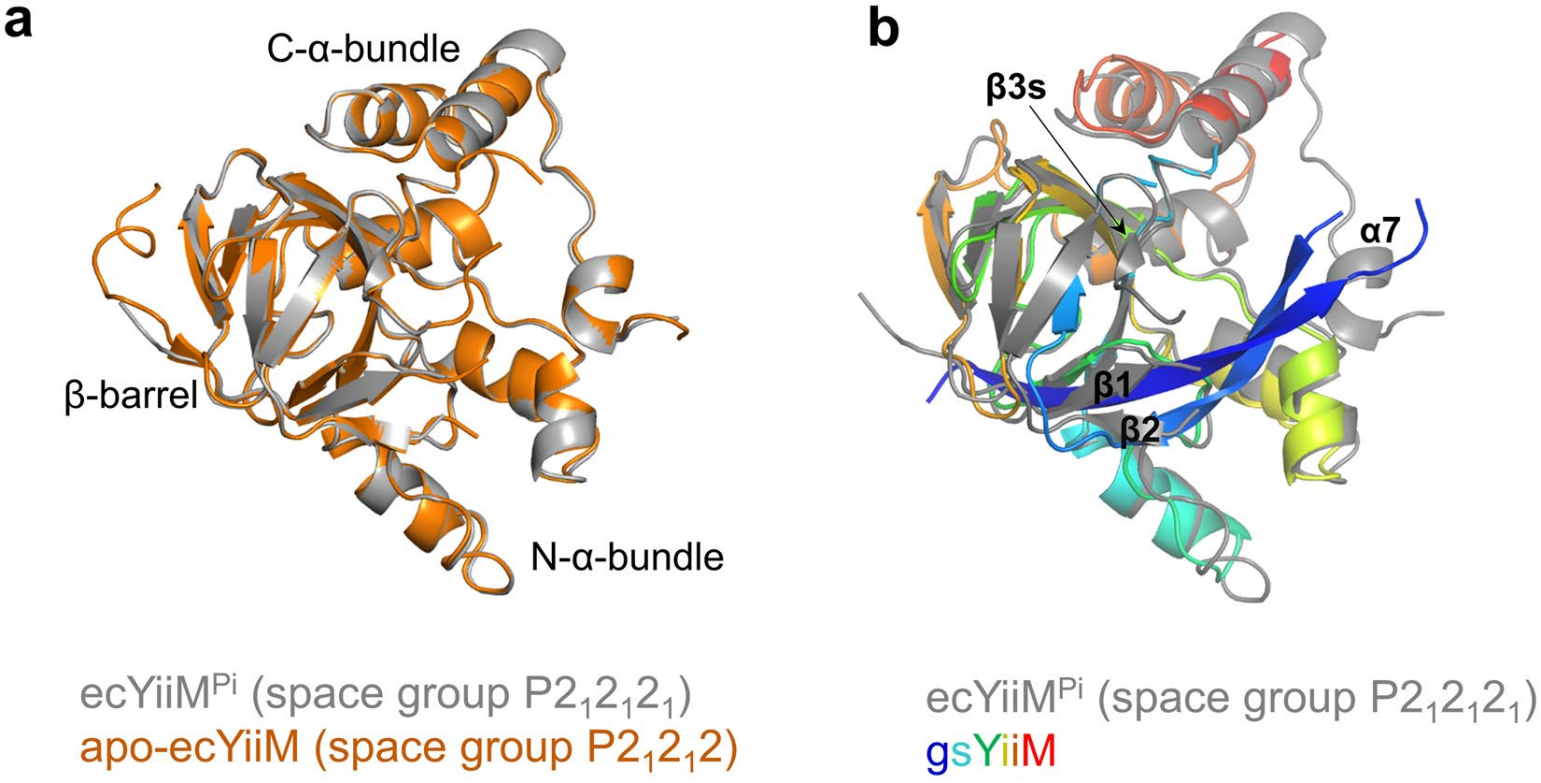
Overlays of YiiM structures. (**a**) Similar structures of ecYiiM. Our ecYiiM^Pi^ structure (space group P2_1_2_1_2_1_) and a previously determined apo-ecYiiM structure (space group P2_1_2_1_2, PDB ID 1O65) are shown as gray and orange ribbons, respectively, in the same orientation as the bottom figure of Fig. 1a. (**b**) Structural comparison of gsYiiM (rainbow-colored ribbons) and ecYiiM^Pi^ (space group P2_1_2_1_2_1_; gray ribbons).

To visualize the Moco-binding mode of YiiM in the catalytic cavity, we attempted to obtain a Moco-conjugated YiiM structure by crystallizing the TP1000-expressed YiiM protein. Although the crystal structure of the TP1000-expressed ecYiiM protein was solved by molecular replacement and refined to 2.85 Å resolution, the electron density for Moco was not visible and instead a phosphate ion was identified (Fig. 4b and Table 1). Notably, our ecYiiM-phosphate complex structure (ecYiiM^Pi^; space group P2_1_2_1_2_1_; a = 56.16 Å, b = 84.84 Å, c = 107.63 Å) differs from a previously reported apo-ecYiiM structure (PDB ID 1O65, space group P2_1_2_1_2, a = 72.12 Å, b = 97.88 Å, c = 98.67 Å) in the crystal system (Table 1)^13^. The ecYiiM^Pi^ and apo-ecYiiM structures are essentially identical, with root-mean-square deviation (RMSD) values of 0.35–0.47 Å (Fig. 5a). ecYiiM exhibits a similar overall structure to gsYiiM but with significant local structural differences (RMSD 1.20–1.24 Å) (Figs 1b and 5b). ecYiiM possesses shorter β1 and β2 strands than gsYiiM. Moreover, ecYiiM contains an additional β-strand (β3s), which functions as a lid on the β-barrel along with the β3 and β9 strands. ecYiiM is also characterized by an additional C-terminal α-helix, α7, which folds back to the N-α-bundle.

Interestingly, in the ecYiiM^Pi^ structure, a phosphate ion was found in the cavity of ecYiiM near the invariant cysteine residue, C120 (Fig. 4b). The phosphate ion in the ecYiiM^Pi^ structure appeared to be captured from crystallization solution containing ammonium dihydrogen phosphate. However, the phosphate ion in the cavity serves as a guide to locate a Moco molecule in the YiiM structure because Moco contains a phosphate group at one end. The phosphate ion in the ecYiiM^Pi^ structure is stabilized by multiple hydrogen bonds with the side chains of cavity residues (Q115, S118, S200, and W201) and partially fills the cavity, leaving space for the remaining atoms of the Moco molecule in the putative catalytic site.

To further define the Moco-binding mode of YiiM in the cavity, we performed *in silico* docking using the ecYiiM^Pi^ structure and selected the best solution in which the phosphate group of Moco is positioned at the phosphate-binding site of the ecYiiM^Pi^ structure (Fig. 4c)^18^. In the Moco-docked ecYiiM model, Moco fits well into the cavity of ecYiiM, with good chemical and shape complementarity. Two ends of Moco, a phosphate group and a pterin ring, are specifically recognized by ecYiiM cavity residues through hydrogen bonds. The phosphate group of the Moco molecule forms multiple hydrogen bonds with Q115, S118, S200, W201, and R55, similar to the phosphate ion observed in the ecYiiM^Pi^ structure. The pterin ring of Moco interacts with A56, N87, and R117 using hydrogen bonds. The invariant cysteine residue (ecYiiM C120) is located near the molybdenum atom of Moco in the ecYiiM-Moco model, suggesting that the invariant cysteine residue plays a key role in the catalytic reaction of the MOSC family.

### Different oligomeric states of gsYiiM and ecYiiM

**Figure 6:**
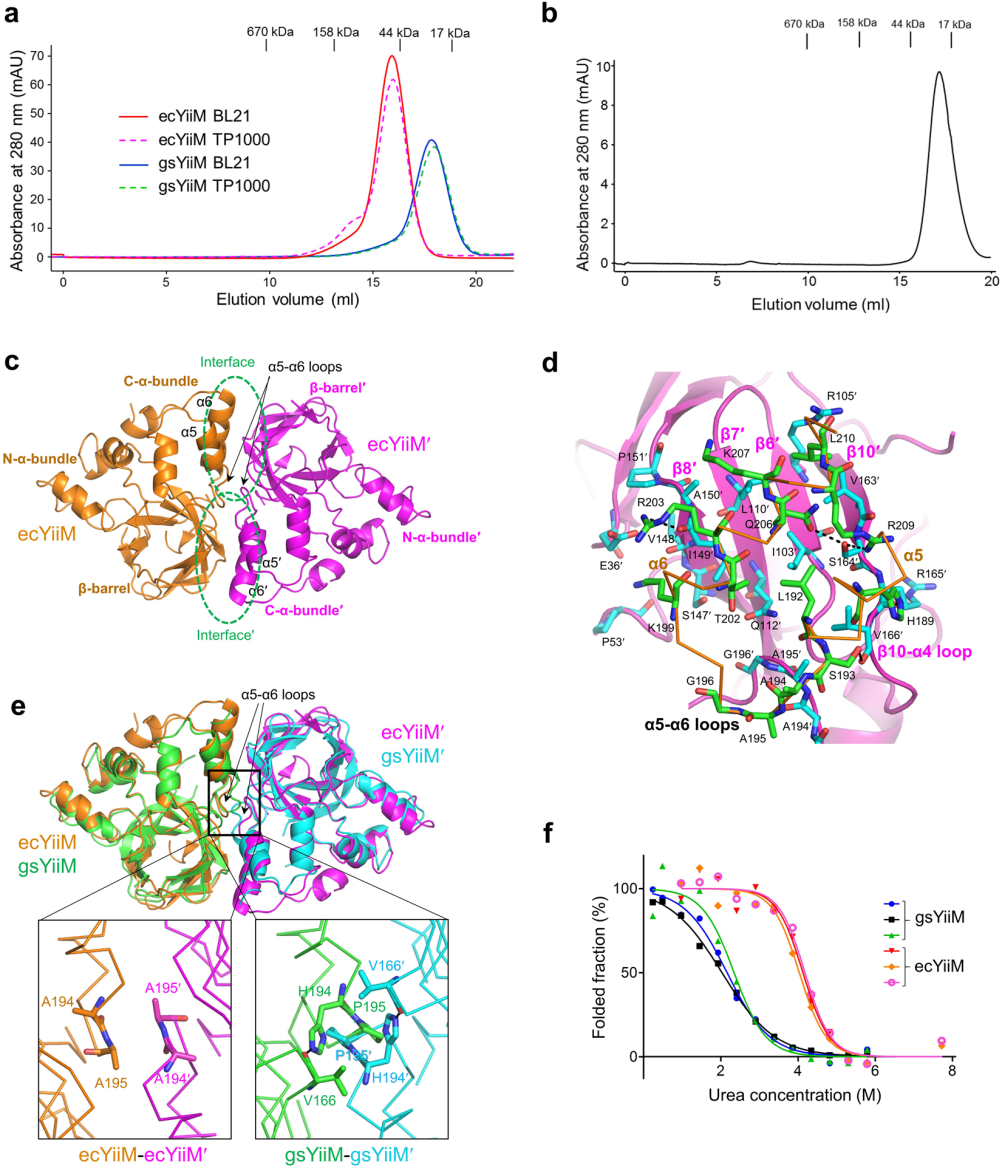
Oligomeric states of gsYiiM and ecYiiM. (**a**) Gel-filtration chromatography to analyze the oligomeric states of ecYiiM and gsYiiM. Both BL21-expressed and TP1000-expressed gsYiiM proteins (calculated molecular weight, 24.1 kDa) were eluted immediately before the 17-kDa protein standard, suggesting that gsYiiM is monomeric in solution. gsYiiM protein that was incubated at 60 °C for 30 minutes was also eluted as a monomer peak (Fig. 6b). In contrast, both BL21-expressed and TP1000-expressed ecYiiM proteins (calculated molecular weight, 25.9 kDa) were eluted immediately before the 44-kDa protein standard, suggesting that ecYiiM is dimeric in solution. (**b**) Gel-filtration chromatography elution profile for the TP1000-expressed gsYiiM protein that was heat-treated at 60 °C for 30 minutes. The heat-treated gsYiiM protein was also eluted as a monomer as was the untreated gsYiiM protein. (**c**) Dimeric ecYiiM^Pi^ structure. The two subunits of the ecYiiM dimer are shown as differently colored ribbons. Two symmetrical dimerization interfaces (labeled “interface” and “interfaceʹ”) are circled in green. (**d**) Dimerization interface of the ecYiiM^Pi^ structure. ecYiiM and ecYiiMʹ chains are shown as orange Cα traces and magenta ribbons, respectively. The dimerization interface residues of ecYiiM and ecYiiMʹ are represented by green and cyan sticks, respectively. (**e**) Overlays of the gsYiiM monomers (green and cyan) on each chain of the ecYiiM^Pi^ dimer (orange and magenta). (**f**) Tryptophan-based protein denaturation profiles of gsYiiM and ecYiiM in the presence of urea denaturant. Three independent measurements were performed for each of the gsYiiM and ecYiiM proteins.

**Figure 7:**
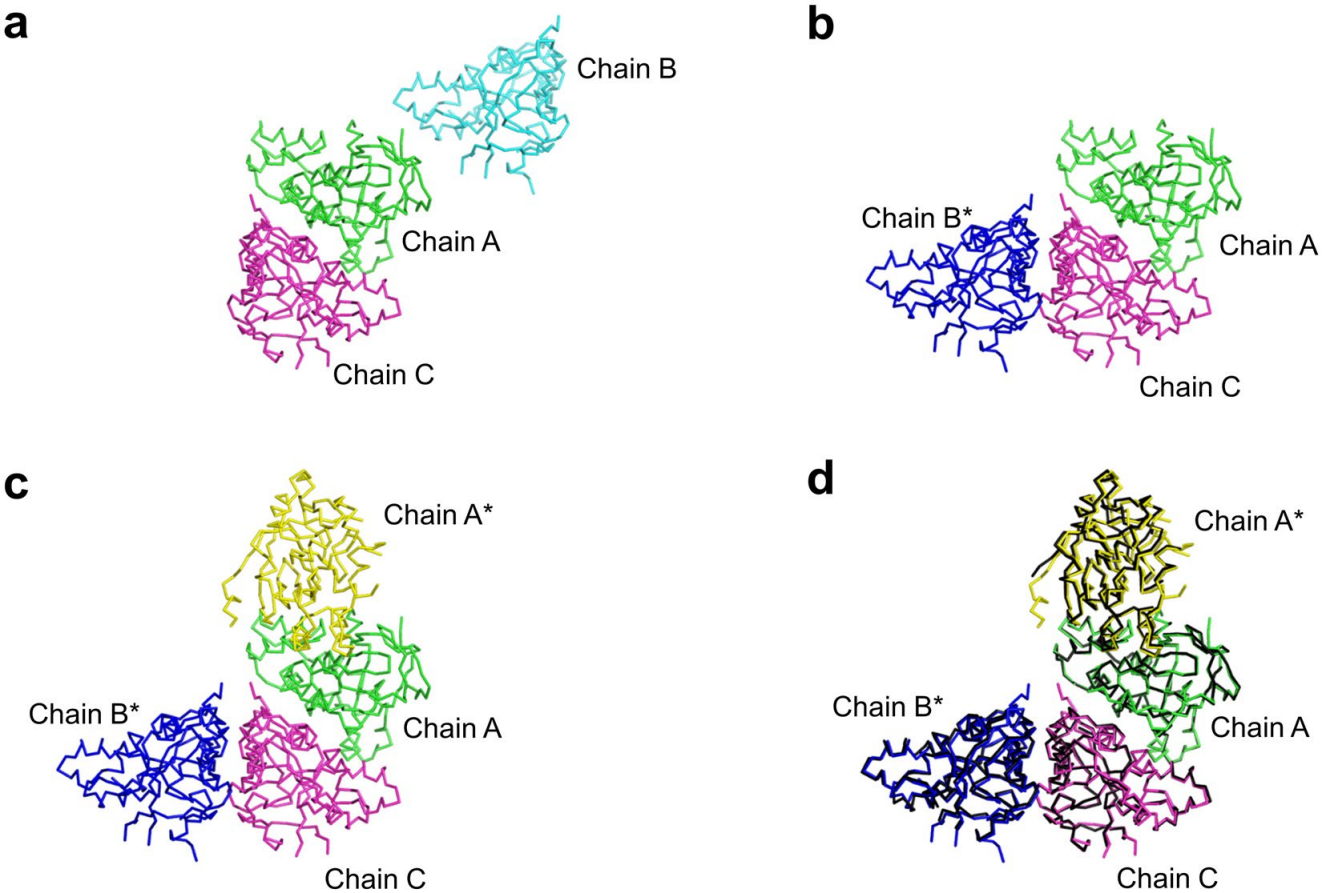
Dimeric organization of ecYiiM observed in the ecYiiM^Pi^ and apo-ecYiiM (PDB ID 1O65) structures. (**a**) Three ecYiiM chains (chain A, green; chain B, cyan; and chain C, magenta) in one asymmetric unit of the apo-ecYiiM structure. (**b**) Rearranged ecYiiM chains in the apo-ecYiiM crystal. Chain B in Fig. 7a was replaced with its symmetry-related molecule (chain B*). Chain B* and chain C form a dimer. (**c**) Dimeric assembly between chain A and its symmetry-related molecule (chain A*, yellow) in the apo-ecYiiM crystal. Chain A* was added into Fig. 7b. (**d**) Identical dimeric organization of the apo-ecYiiM and ecYiiM^Pi^ structures. Two ecYiiM^Pi^ dimers (black) are overlaid on the A-A* dimer and B*-C dimer of the apo-ecYiiM structure.

The high sequence and shape conservation of the gsYiiM and ecYiiM cavities suggest that gsYiiM and ecYiiM exert nitrogen-reduction activity through a common enzymatic reaction mechanism that employs the putative catalytic cavity (Figs 2a,b and 4d). Nonetheless, gsYiiM and ecYiiM enzymes seem to have differently evolved biophysical properties, such as oligomeric state and protein stability (Fig. 6). gsYiiM and ecYiiM adopt monomeric and dimeric forms, respectively, both in solution and in crystal (Figs 1a and 6a,b,c). In gel-filtration chromatography, both the BL21-expressed and TP1000-expressed gsYiiM proteins were eluted as monomers (Fig. 6a,b). Additionally, gsYiiM was observed as a monomer in the asymmetric unit without any suggestive oligomerization partner (Fig. 1a). In contrast to gsYiiM, ecYiiM was eluted as a dimer in the gel-filtration chromatography irrespective of the protein expression system (Fig. 6a). Consistently, the asymmetric unit of the ecYiiM^Pi^ crystal contained two ecYiiM chains, which are related by non-crystallographic two-fold symmetry (Fig. 6c). The dimeric organization of the ecYiiM^Pi^ structure is also recapitulated in the apo-ecYiiM structure (PDB ID 1O65) (Fig. 7).

Upon dimerization, each ecYiiM chain buries a surface area of ~1,090 Å^2^ into two symmetrical binding interfaces (labeled “interface” and “interfaceʹ” in Fig. 6c; the prime denotes the dimerization partner). In each interface, the α5 and α6 helices of the C-α-bundle from one subunit make contacts with one side (β8′-β7′-β6′-β10′) of the β-barrel from the other subunit. The dimerization interface is characterized by hydrophobic and van der Waals interactions in the center, which is surrounded by hydrophilic interactions, including ten hydrogen bonds (Fig. 6d). The ecYiiM dimer is further fastened by symmetrical van der Waals interactions between the α5-α6/β10-α4 loops and α5ʹ-α6ʹ/β10ʹ-α4ʹ loops at the boundary of the two symmetrical interfaces. The α5-α6 loop appears to play a key role in determining the oligomeric state of YiiM. The α5-α6 loop in the ecYiiM^Pi^ structure adopts a relatively flat surface (Fig. 6e). In contrast, the α5-α6 loop of gsYiiM protrudes and makes significant steric clashes with the α5ʹ-α6ʹ loop in the dimeric organization, providing an explanation why gsYiiM prefers a monomeric form although the dimerization interface residues of ecYiiM are largely conserved in gsYiiM (Figs 3 and 6e). The protrusion of the α5-α6 loop seems to be caused by gsYiiM H194 and P195 (Fig. 6e). The bulky side chain of gsYiiM H194 is inserted under the β10-α4 loop through a stable hydrogen bond with the V166 main chain of the β10-α4 loop, allowing the α5-α6 loop to be hoisted. gsYiiM P195 appears to stabilize the α5-α6 loop into the protruding conformation though the proline-specific rigidity in the main chain. In ecYiiM, the H194 and P195 residues of gsYiiM are replaced with alanine residues (A194 and A195) (Fig. 3).

Dimerization buries a large surface of ecYiiM, including hydrophobic regions, in the binding interface, suggesting that ecYiiM dimerization is required to stabilize protein. To address the contribution of dimerization to the protein stability of YiiM, we monitored the denaturation profiles of gsYiiM and ecYiiM proteins by measuring tryptophan fluorescence intensity in the presence of a protein denaturant, urea. ecYiiM protein was highly stable against urea, with a denaturant concentration at half denaturation (C_1/2_) of 4.11 ± 0.07 M (Fig. 6f). However, gsYiiM exhibited substantially lower stability (C_1/2_, 2.18 ± 0.16 M) than ecYiiM. The stability difference between ecYiiM and gsYiiM can be ascribed to their distinct oligomeric states (the dimeric form of ecYiiM versus the monomeric form of gsYiiM). It is unexpected that ecYiiM would have higher intrinsic stability than gsYiiM because proteins from thermophilic microorganisms, such as *G. stearothermophilus*, are generally more stable than proteins from mesophiles, such as *E. coli*. Moreover, dimerization would affect the catalytic activity of YiiM because the α6 helix is not only involved in the dimerization but also contributes to the formation of the putative catalytic cavity.

### Structural comparison of YiiM with its structural homologs

**Figure 8:**
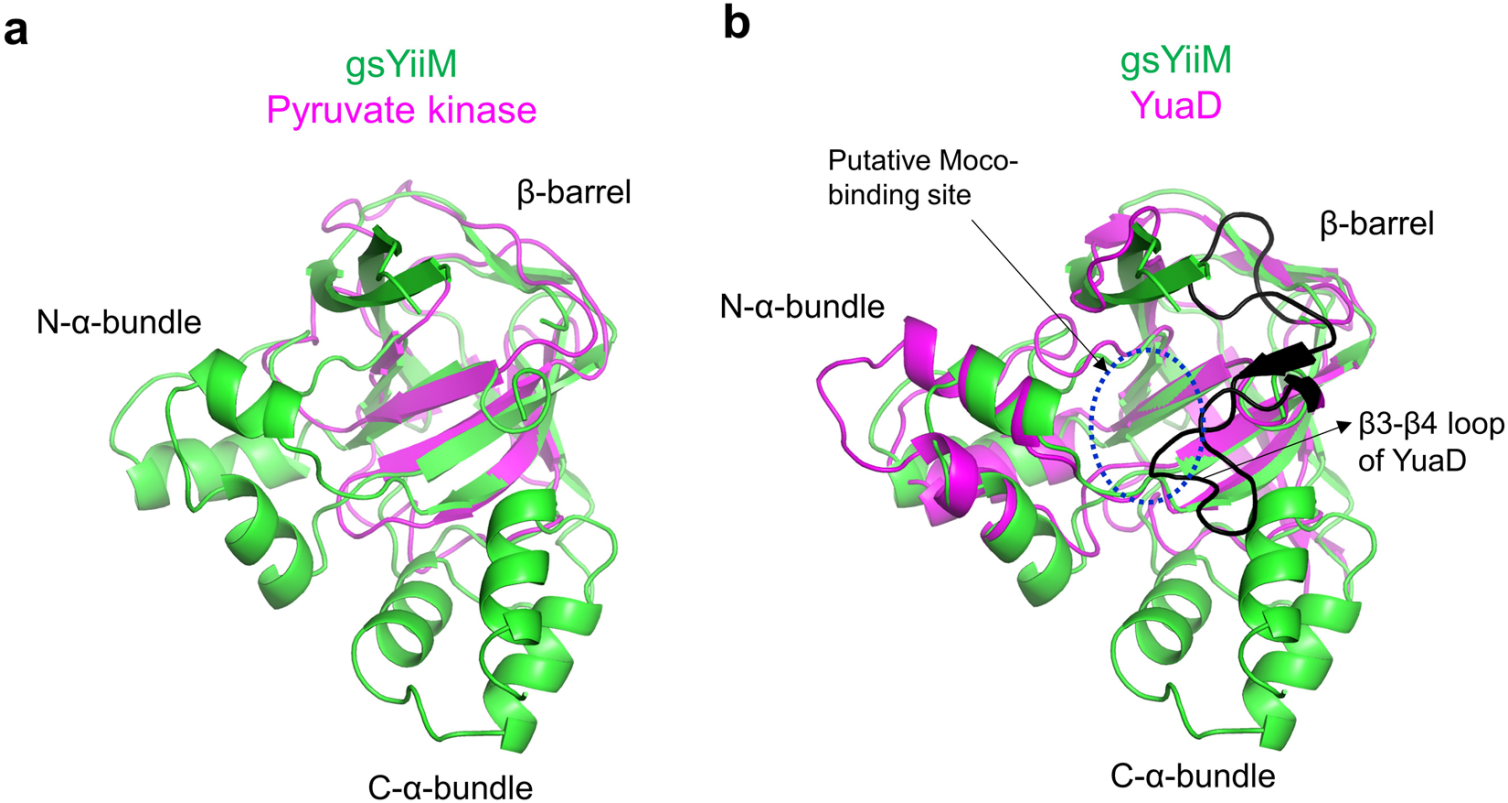
Structural comparison of YiiM with its homologs. (**a**) gsYiiM (green ribbons) and the domain B of pyruvate kinase (PDB ID 1A49, magenta ribbons). (**b**) gsYiiM (green ribbons) and YuaD (PDB ID 1ORU, magenta ribbons). The extended β3-β4 loop of YuaD is colored in black. The putative Moco-binding site of YiiM is circled in a blue dotted line.

A Dali search indicated that YiiM is structurally homologous to pyruvate kinase and the uncharacterized YuaD protein (Fig. 8)^19^. The β-barrel region of the gsYiiM structure can be overlaid on that of the domain B of pyruvate kinase (PDB ID 1A49), with an RMSD value of 1.95 Å for 82 Cα atoms (Fig. 8a)^20^. Despite the structural similarity, the β-barrels of YiiM and pyruvate kinase are functionally unrelated. The β-barrel of pyruvate kinase acts as a lid to regulate the accessibility of substrate to a catalytic site located in other domains of pyruvate kinase. Moreover, the β-barrel of the pyruvate kinase is not linked to N-terminal or C-terminal α-helix bundles, which are required to constitute a putative catalytic site in YiiM.

The gsYiiM structure is closely related to the unpublished structure of YuaD (PDB ID 1ORU), which was determined by the Midwest Center for Structural Genomics (Fig. 8b). Sequence analysis indicates that YuaD belongs to the MOSC family although its function has not been characterized^4^. YuaD displays a β-barrel and an N-α-bundle that are structurally comparable to those of gsYiiM with an RMSD value of 1.96 Å for 137 Cα atoms, suggesting that the MOSC family evolved from a common ancestral MOSC domain containing a β-barrel and an α-helix bundle. However, unlike YiiM, YuaD does not possess the C-α-bundle and is characterized by an elongated β3-β4 loop (35 residues in YuaD vs 22 residues in YiiM) that includes two additional β-strands. Interestingly, the structural overlays of YiiM and YuaD indicate that the protruding β3-β4 loop of YuaD replaces the C-α-bundle of YiiM and forms a putative Moco-binding site. In other MOSC proteins, including mARC and YcbX, a β-barrel-like sequence is commonly found in addition to the MOSC domain and seems to be the functional counterpart of the C-α-bundle of YiiM. Future structural studies of other MOSC members are required to reveal the exact Moco-binding site and the catalytic mechanism of the MOSC family and to develop a novel anti-bacterial drug.

## Methods

### Construction of YiiM expression vectors

The gsYiiM and ecYiiM genes were amplified by polymerase chain reaction (PCR) using the template of *G. stearothermophilus* and *E. coli* genomic DNAs to construct the gsYiiM and ecYiiM expression plasmids, respectively. Primers were designed to possess B*am*HI and *Sal*I restriction enzyme sites at the distal ends of PCR products. The PCR products were digested using *Bam*HI and *Sal*I, and the resulting fragments were ligated into modified pET49b or pQE80L expression plasmids that contain an N-terminal His_6_ tag and a thrombin cleavage site^21^. The ligation product was transformed into *E. coli* strain DH5α. pET49b and pQE80L transformants were selected on LB-kanamycin and LB-ampicillin agar plates, respectively, and their nucleotide sequences were confirmed by DNA sequencing.

### Protein expression and purification

For recombinant protein expression of gsYiiM and ecYiiM, pET49b and pQE80L plasmid DNAs containing the YiiM gene were transformed into *E. coli* strains BL21 (DE3) and TP1000^16,22^. The BL21 (DE3) and TP1000 cells were grown in 600 ml of LB-kanamycin and LB-ampicillin media, respectively, at 37 °C. Protein expression was induced overnight at 18 °C in BL21 (DE3) cells by adding 1 mM IPTG when the optical density of the culture at 600 nm reached ~0.7. Protein expression in TP1000 cells was induced by adding 1 mM sodium molybdate and 15 μM IPTG at an optical density of ∼0.1 at 600 nm, and the cells were then grown at 20 °C overnight.

The cells were harvested by centrifugation and resuspended in a lysis buffer (50 mM Tris, pH 8.0, 200 mM NaCl, 5 mM β-mercaptoethanol, and 1 mM PMSF). The resultant cells were homogenized using a sonicator. The lysate was cleared by centrifugation, and the supernatant was incubated with Ni-NTA resin. The mixture was loaded onto a glass Econo-Column and washed using a solution containing 50 mM Tris, pH 8.0, 200 mM NaCl, 5 mM β-mercaptoethanol, and 10 mM imidazole. YiiM protein was eluted using a solution containing 50 mM Tris, pH 8.0, 200 mM NaCl, 5 mM β-mercaptoethanol, and 250 mM imidazole. The purified gsYiiM and ecYiiM proteins were dialyzed against 20 mM Tris, pH 8.5, and 20 mM Tris, pH 8.0, respectively, in the presence of 5 mM β-mercaptoethanol, and the N-terminal His_6_ tag was removed by thrombin. gsYiiM was further purified by anion-exchange chromatography. The tag-free gsYiiM protein was injected into a mono Q 10/100 column that had been equilibrated in 20 mM Tris, pH 8.5, and 5 mM β-mercaptoethanol, and gsYiiM was eluted using a linear NaCl gradient (0–500 mM) in 20 mM Tris, pH 8.5, and 5 mM β-mercaptoethanol. For ecYiiM, gel-filtration chromatography was employed as the second purification step after the Ni-NTA affinity chromatography and thrombin digestion. The ecYiiM protein was injected into a Superdex 200 10/600 column in 20 mM Tris, pH 8.0, 150 mM NaCl, and 5 mM β-mercaptoethanol. Chromatography fractions corresponding to YiiM protein were collected and concentrated for crystallization. The oligomeric states of gsYiiM and ecYiiM were analyzed in solution by gel-filtration chromatography at room temperature using a Superdex 200 10/300 column in a solution containing 20 mM Tris, pH 8.0, 150 mM NaCl, and 5 mM β-mercaptoethanol.

### Crystallization

Crystallization conditions for YiiM were screened using the JCSG Core Suites kit (Qiagen) by the sitting-drop vapor-diffusion method at 18 °C. Crystals of the BL21-expressed gsYiiM protein were obtained in a drop consisting of 0.5 μl of 12.7 mg/ml gsYiiM in 20 mM Tris, pH 8.5, 150 mM NaCl, and 5 mM β-mercaptoethanol and 0.5 μl of a solution containing 0.095 M sodium citrate, pH 5.6, 21% PEG 4000, 19% isopropanol, and 5% glycerol. The TP1000-expressed ecYiiM protein was crystallized in a drop that contained 0.5 μl of 8.5 mg/ml ecYiiM in 20 mM Tris, pH 8.0, 150 mM NaCl, and 5 mM β-mercaptoethanol and 0.5 μl of a solution of 1.1 M ammonium dihydrogen phosphate, 0.08 M CHES, pH 9.5, and 20% glycerol.

### Data collection and processing

X-ray diffraction was performed at beamlines 5 C and 7 A of the Pohang Accelerator Laboratory (PAL, Republic of Korea). Crystals were cryoprotected using 25% glycerol. Crystals were harvested by a nylon loop and flash-cooled under cryo-stream. X-ray diffraction data were collected with 0.5° oscillation. Diffraction data were reduced and scaled using the HKL-2000 package^23^. X-ray diffraction statistics are shown in Table 1.

### Structure solution and refinement

The crystal structures of gsYiiM and ecYiiM were solved by molecular replacement, which was performed with the PHASER program^24^ using a search model of the ecYiiM structure (PDB ID 1O65) that had been determined through a bacterial structural genomics project^13^. Iterative model building and refinement were performed using the COOT and REFMAC5 programs, respectively^25,26^. The structure refinement statistics are shown in Table 1.

### Tryptophan fluorescence-based protein denaturation assay

A tryptophan fluorescence-based protein denaturation assay was performed to determine the protein stability of YiiM. The BL21-expressed YiiM protein was incubated in 20 mM Tris, pH 8.0, and 150 mM NaCl in the presence of urea (ecYiiM, 0.97–7.72 M; gsYiiM, 0.24–5.79 M) at room temperature for 30 min. The intensity of tryptophan fluorescence emitted at 325 nm was measured using a Synergy H1 instrument (BioTek) with an excitation wavelength of 295 nm.

### ICP-MS

The molybdenum content of the YiiM protein was analyzed by ICP-MS. For the analysis, YiiM protein samples were pretreated by incubating the protein (2 ml) with nitric acid (6 ml) at 180 °C for three hours. The digests were mixed with water (15 ml), decanted at 185 °C, and re-filled with water to a final volume of 20 ml. 1 ml of the resulting sample was analyzed by ICP-MS using the NexION 350D instrument (PerkinElmer) to detect the molybdenum masses 92, 95, 96, 97, and 98.

### Data deposition

The atomic coordinates and structure factors for YiiM (PDB ID 5YHH and 5YHI) have been deposited in the Protein Data Bank, www.pdb.org.
